# Isoform-selective activity-based profiling of ERK signaling†
†Electronic supplementary information (ESI) available: Experimental Methods and Supplementary Figures. See DOI: 10.1039/c8sc00043c


**DOI:** 10.1039/c8sc00043c

**Published:** 2018-02-06

**Authors:** Myungsun Shin, Caroline E. Franks, Ku-Lung Hsu

**Affiliations:** a Department of Chemistry , University of Virginia , McCormick Road, P.O. Box 400319 , Charlottesville , Virginia 22904 , USA . Email: kenhsu@virginia.edu ; Tel: +1-434-297-4864; b Department of Pharmacology , University of Virginia , McCormick Road, P.O. Box 400319 , Charlottesville , Virginia 22908 , USA; c University of Virginia Cancer Center , University of Virginia , Charlottesville , VA 22903 , USA

## Abstract

Extracellular signal-regulated kinases (ERKs) mediate downstream signaling of RAS-RAF-MEK as key regulators of the mitogen-activated protein kinase (MAPK) pathway. Activation of ERK signaling is a hallmark of cancer and upstream MAPK proteins have been extensively pursued as drug targets for cancer therapies. However, the rapid rise of resistance to clinical RAF and MEK inhibitors has prompted interest in targeting ERK (ERK1 and ERK2 isoforms) directly for cancer therapy. Current methods for evaluating activity of inhibitors against ERK isoforms are based primarily on analysis of recombinant proteins. Strategies to directly and independently profile native ERK1 and ERK2 activity would greatly complement current cell biological tools used to probe and target ERK function. Here, we present a quantitative chemoproteomic strategy that utilizes active-site directed probes to directly quantify native ERK activity in an isoform-specific fashion. We exploit a single isoleucine/leucine difference in ERK substrate binding sites to enable activity-based profiling of ERK1 *versus* ERK2 across a variety of cell types, tissues, and species. We used our chemoproteomic strategy to determine potency and selectivity of academic (VX-11e) and clinical (Ulixertinib) ERK inhibitors. Correlation of potency estimates by chemoproteomics with anti-proliferative activity of VX-11e and Ulixertinib revealed that >90% inactivation of both native ERK1 and ERK2 is needed to mediate cellular activity of inhibitors. Our findings introduce one of the first assays capable of independent evaluation of native ERK1 and ERK2 activity to advance drug discovery of oncogenic MAPK pathways.

## Abstract 2

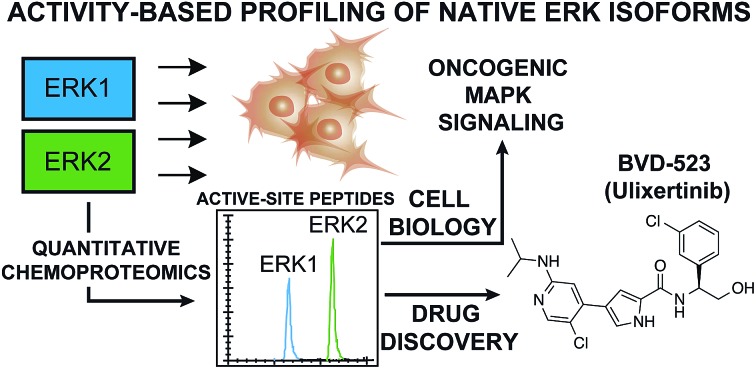
We report a novel chemical biology approach to study isoform-specific activity of extracellular signal-regulated kinases (ERKs), which are key regulators of signal transduction and emerging drug targets for oncology.

## Introduction

**Fig. 1:**
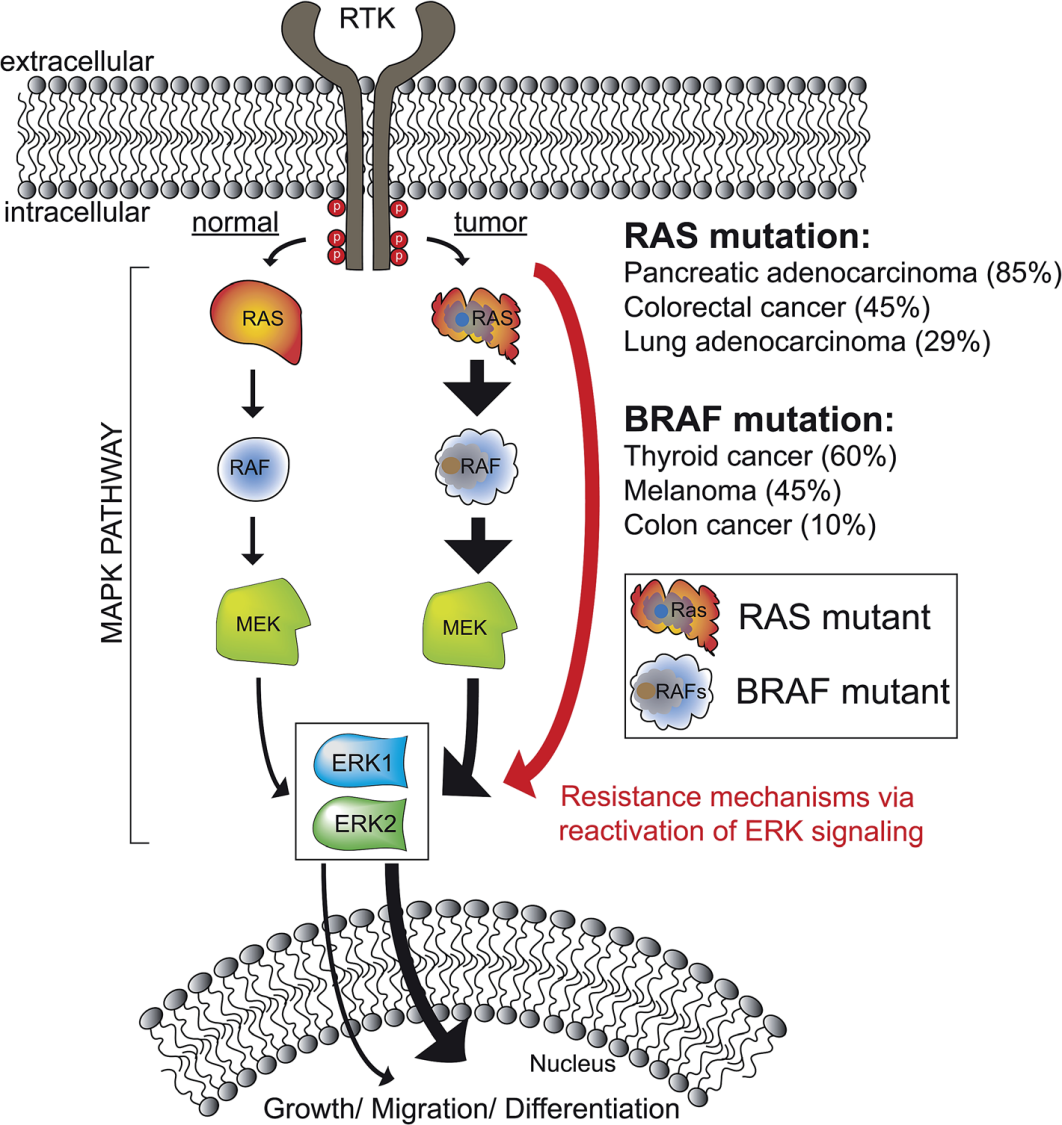
The ERK MAPK signaling pathway. Kinases mediating ERK MAPK signaling are sequentially activated by phosphorylation. ERK1/2 are terminal kinases in MAPK signaling that can translocate to the nucleus to regulate transcription programs mediating growth/migration/differentiation. Aberrant activation of ERK signaling through RAS and RAF mutations is observed in >30% of human cancers and extensive drug discovery efforts have been directed towards this pathway for targeted cancer therapies. However, reactivation of ERK signaling with RAF and MEK inhibitors has prompted interest in targeting ERK1/2 directly. Percentages reflect the TCGA provisional data sets accessed *via* cBioPortal (; http://www.cbioportal.org/).

The mitogen-activated protein kinase (MAPK) pathway is fundamental to cell biology because of its role in integrating cell surface signals to transcriptional regulation of the proteome.^1^–^3^ In the extracellular signal-regulated kinase (ERK) MAPK pathway, growth factors and mitogens trigger activation of receptor tyrosine kinases (RTKs) that mediate guanosine triphosphate (GTP) loading of the RAS GTPase.^4^ GTP-loaded RAS can recruit RAF (ARAF, BRAF, and CRAF) to the cell membrane resulting in activation; activated RAF can phosphorylate and activate MEK (MEK1 and MEK2), which phosphorylates and activates ERK (ERK1 and ERK2) as part of a signaling cascade to modulate cell proliferation, differentiation, apoptosis, and migration^1^,^5^,^6^ (Fig. 1). Mutations that activate the MAPK pathway are found in >30% of human cancers and as a result, efforts to develop drugs against members of the ERK cascade have been extensively pursued.^7^–^9^ Despite initial clinical response using BRAF^10^,^11^ and MEK inhibitors,^12^,^13^ the rapid rise of resistance has limited the durability of BRAF/MEK drugs.^14^ Reactivation of ERK signaling in tumors resistant to BRAF/MEK inhibitors has prompted interest in targeting these downstream kinases directly for cancer therapy.^15^,^16^

As a central mediator of the MAPK pathway (Fig. 1), ERKs fine-tune cellular responses through phosphorylation of diverse substrates to modulate transcriptional programs.^6^ To date, >200 putative ERK substrates found in both nuclear and cytoplasmic locales have been identified from global proteomic studies.^17^ The wide substrate profile of ERKs has led to questions as to how the MAPK pathway regulates specific biological responses.^18^ Debate remains in the field as to whether ERK1 and ERK2 exhibit overlapping or distinct biological functions.^19^,^20^ The high sequence homology (>80% identity^6^,^15^), *in vitro* evidence of equivalent catalytic activity,^21^ and seemingly parallel activation of ERK1 and ERK2 in cellular systems support functional redundancy.^22^ However, other groups have reported ERK1 (ref. 23 and 24) and ERK2-specific functions in several biological systems.^25^–^27^ Genetic knockout models further support that ERKs are not functionally redundant; ERK2 ablation is embryonic lethal^28^,^29^ while ERK1 knockout mice are viable and fertile.^30^ One of the challenges impeding testing of ERK isoform-specific functions is the lack of assays capable of direct measurement of endogenous ERK1 *versus* ERK2 activity.

**Fig. 2:**
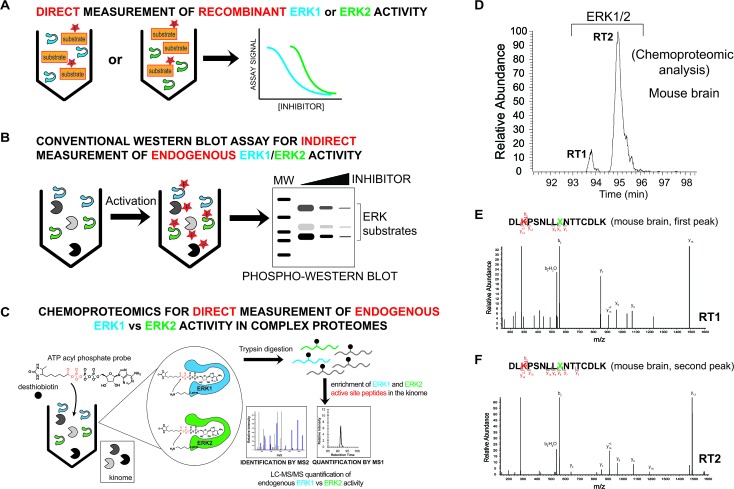
Development of a chemoproteomic assay for direct and isoform-specific evaluation of native ERK activity. (A) Biochemical assays for measuring recombinant ERK1 and ERK2 activity. Substrate assays can measure individual ERK isoforms but are limited to assaying of recombinant proteins. (B) Western blots can measure native ERK1/2 activity but readouts are indirect and often cannot discern isoform specificity of inhibitors. Phosphorylation of ERK1/2 and downstream substrates are biomarkers used to evaluate cellular activity of compounds. (C) Schematic of a chemoproteomic assay to measure native ERK activity in an isoform-specific fashion. Measurement of ERK1 and ERK2 in complex proteomes enables parallel evaluation of potency and selectivity. Conserved lysines in the active-sites of kinases react with acyl phosphate groups of ATP activity-based probes to covalently modify active kinases with desthiobiotin tags for quantitative LC-MS/MS analysis. (D) MS1 extracted ion chromatogram (EIC) of a peptide *m*/*z* from chemoproteomic analysis of mouse brain proteomes resulted in ambiguous identification of 2 LC-resolved peptides that matched ERK1/2 (labeled RT1 and RT2). (E, F) MS2 spectra resulting from fragmentation of the same peptide *m*/*z* for each LC peak (RT1 and RT2). The spectra showed an identical fragmentation pattern from each chromatographically separated peak. Red amino acid indicates probe-modified lysine residue. Green X indicates an ambiguous isoleucine/leucine isomer in ERK active-site peptides that cannot be distinguished from MS2 spectra.

Current methods for measuring ERK activity consist mainly of biochemical assays using purified recombinant ERK1 and ERK2 to measure substrate specificity and inhibitor activity^31^ (Fig. 2A). More advanced screening platforms (*e.g.* KINOMEscan®) take advantage of recombinant ERK fusion proteins with T7 bacteriophage to enable rapid evaluation of inhibitor activity in lysates.^32^,^33^ ERKs are activated by phosphorylation and phosphorylated ERK1 and ERK2 (phospho-ERK1/2) serve as widely-used biomarkers of native ERK activity. Phospho-ERK1/2 is often combined with measurements of phosphorylated downstream substrates (*e.g.* p90RSK) to monitor ERK activity and inhibition in cell biological assays^6^,^34^ (Fig. 2B). Additional methods have been used for ERK analysis including genetically encoded FRET sensors,^35^ NMR spectroscopy,^36^ and covalent probes.^37^,^38^ To the best of our knowledge, no method has demonstrated the ability to evaluate inhibition of endogenous ERKs (*i.e.* ERK1 and ERK2) with isoform specificity in complex proteomes.

Here, we present a chemoproteomic strategy for direct evaluation of native ERK1 *versus* ERK2 activity in complex proteomes. We used ATP acyl phosphate activity-based probes^39^,^40^ and quantitative mass spectrometry^41^,^42^ to survey ERK active-sites for features that enable isoform-selective profiling. We addressed challenges with studying the near-identical substrate binding pocket^20^ of ERK isoforms by revealing a single mass-indistinguishable isoleucine/leucine (I/L) difference that could be exploited for quantitation by chemoproteomics. We synthesized peptides corresponding to active-site regions containing the I/L isomer to recapitulate the chromatographic phenomenon (*i.e.* elution profile) and validate isoform specificity of our activity-based profiling assay of native ERK1 and ERK2. We used our assay to determine isoform selectivity of academic (VX-11e) and clinical (Ulixertinib) ERK inhibitors to discover that chemoproteomic inhibitor binding profiles are more predictive of compound activity against tumor cells compared with traditional cell biological readouts (*i.e.* phospho-ERK1/2). Given that several ERK inhibitors are in clinical trials,^15^,^43^,^44^ our assay and findings should be of utility for guiding efforts to target ERK isoforms in oncology.

## Experimental section

### Materials

Reagents used were purchased from Fisher Scientific unless specified otherwise. Primary antibodies were purchased from Cell-Signaling Technologies: phospho-p44/42 MAPK (Erk1/2) (Thr202/Tyr204) Antibody (catalog # 9101S); phospho-p90RSK (Thr359/Ser363) Antibody (catalog # 9344S). Secondary fluorescent conjugated antibodies were obtained from Thermo Fisher Scientific (goat anti-Rabbit-DyLight 550, catalog # 84541). Fetal bovine serum (FBS), and dialyzed fetal bovine serum (dialyzed-FBS) were obtained from Omega Scientific. Ulixertinib (BVD-523, catalog # S7854) and VX-11e (catalog # S7709) were obtained from Selleckchem.

### Cell culture

A549, H82, H1650, and DM122 cells were maintained in RPMI 1640 (Fisher Scientific) and HEK293T cells were maintained in DMEM (Fisher Scientific) supplemented with 10% FBS (Omega Scientific, US Source Fetal Bovine Serum), 2 mM l-glutamine (Thermo Fisher Scientific), 10 units and 100 μg mL^–1^ penicillin–streptomycin (Sigma-Aldrich). SILAC A549 and HEK293T cells were maintained in RPMI 1640 and DMEM media, respectively, for SILAC without l-lysine and l-arginine (Thermo Scientific) supplemented with 10% dialyzed FBS (Omega Scientific, US Source Fetal Bovine Serum), 2 mM l-glutamine (Thermo Fisher Scientific), 10 units and 100 μg mL^–1^ penicillin–streptomycin (Sigma-Aldrich). ‘Light SILAC’ media was supplemented with l-lysine and l-arginine (100 μg mL^–1^, Acros Organics). ‘Heavy SILAC’ media was supplemented with isotopically labeled l-lysine (^13^C_6_, ^15^N_2_) and l-arginine (^13^C_6_, ^15^N_4_, 100 μg mL^–1^, Sigma-Aldrich) for a minimum of five passages prior to use. Cells were cultured at 37 °C in 5% CO_2_.

### Biological tissues

Mouse brains were obtained from C57BL/6 mice. Mice were anesthetized with isoflurane (Henry Schein Animal Health) and sacrificed by cervical dislocation. Collected tissues were washed with PBS before snap freezing in liquid nitrogen. Brain tissue from Zebra finch (*Taeniopygia guttata*) was gifted by Dr Daniel Meliza (Department of Biology, University of Virginia). All animal experiments were conducted in accordance with the guidelines of the Institutional Animal Care and Use Committee at the University of Virginia. The experiments performed were approved by the Animal Care and Use Committee at the University of Virginia (animal protocol no. 4034).

### Western blot analysis of p-RSK and p-ERK1/2

Cell lysates were separated *via* centrifugation at 100 000 × *g* for 45 min at 4 °C. Proteins separated by SDS-PAGE (4–20% polyacrylamide, TGX Stain-Free MIDI Gel). Gel transfers were performed using the Bio-Rad Trans-Blot Turbo RTA Midi Nitrocellulose Transfer Kit with a Bio-Rad Trans-Blot Turbo Transfer System. After incubation with 5% milk in TBST (1.5 M NaCl, 0.25 M Tris pH 7.4, 0.1% Tween 20, in ultrapure water (ddH_2_O)) for 1 h. The membrane was incubated with primary antibody p-RSK (1 : 1000), or p-ERK1/2 (1 : 1000) for 12 h at 4 °C. After the primary antibody incubation, membranes were washed 5 times for 5 min with TBST and incubated with secondary antibody (1 : 10 000) for 2 h at 25 °C. The membrane was washed 5 times for 5 min with TBST and imaged with a Chemidoc MP Imaging system.

### WST-1 viability assay

A549 cells were detached from the plate using trypsin and plated in cell culture treated 96 well plate in serum free RPMI 1640. Cells were treated with inhibitors and incubated at 37 °C for 96 h. WST-1 reagent (Roche) was added to each well according to manufacturer's instruction and incubated at 37 °C for 2 h. After the incubation, the absorbance was measured at 450 nm using a CLARIOstar microplate reader (BMG Labtech).

### Synthetic peptides

Peptide with sequences DLKPSNLLLNTTCDLK and DLKPSNLLINTTCDLK were obtained from Atlantic Peptides, with reported purities of 98.86% and 99.09%, respectively. Synthetic peptides were analyzed using a Waters 1525 HPLC with X-bridge C18 5 μm, 4.6 × 150 mm column, with mobile phase (A) 0.1% TFA in ddH2O, and mobile phase (B) 0.1% TFA in CH_3_CN. Gradient used was 10–50% B in 20 min.

### CD4+ T cell expansion

Purified human CD4+ T cells from peripheral blood (1.5 × 10^7^ cells (Stemcell Technologies)) were resuspended in complete RPMI (10% FBS, 1% l-Glut, 1% Pen/Strep) at a concentration of 5 × 10^5^ cells per mL (30 mL). Human T-Activator CD3/CD28 Dynabeads™ (Gibco) were resuspended in the vial by vortexing and the desired volume was transferred to an Eppendorf tube. Beads were rinsed with PBS + 0.1% BSA (1 mL) by vortexing for at least 30 s. Beads were isolated using a magnet and resuspended in the same volume of RPMI culture medium as the initial volume of beads taken from vial. Beads were then added to T cell culture at a ratio of 1 : 1 (375 μL beads for 1.5 × 10^7^ cells). Recombinant human IL-2 was added to T cell culture at a concentration of 30 U per mL. The culture was incubated in 5% CO_2_ at 37 °C for ten days. Cell density was measured daily. When cells reached 2.5 × 10^6^ cells per mL the culture was split to 5 × 10^5^ cells per mL with fresh complete RPMI with 30 U per mL recombinant human IL-2.

### Sample preparation for quantitative LC-MS analysis using ATP acyl phosphates

Samples were prepared for LC-MS analysis as previously described.^41^,^42^ See ESI† for additional details.

### LC-MS/MS analysis of proteomics samples

LC-MS analyses were performed as previously described.^41^,^42^ See ESI† for additional details.

### Modeling of ERK1 and ERK2 crystal structures

Crystal structures of ERK1 (ID 4QTB) and ERK2 (; 4QP4) were obtained from the Protein Data Bank (; https://www.rcsb.org/pdb/home/home.do). Monomeric ERK1 and ERK2 were aligned using PyMol (; https://pymol.org/2/) and the catalytic lysine (ERK1:K168, ERK2: K151) and isoleucine/leucine (ERK1:I174, ERK2:L157) found in active-sites of each isoform were highlighted to generate Fig. 4B.

### Statistical analysis and determination of IC_50_ values

For chemoproteomics analysis, the percentage of enzyme activity was determined from SILAC ratios obtained from LC-MS analyses and normalized to the DMSO control (Light-DMSO, Heavy-DMSO) sample. Dose–response curves of inhibitor concentration (log scale) and % control was used to determine potency (half maximum inhibitory concentration, IC_50_) by fitting curves using nonlinear regression analysis (one site – Fit log IC_50_) in GraphPad Prism. For the WST-1 assay used to generate EC_50_ estimates of cell viability, GraphPad prism software was used to perform nonlinear regression analysis. EC_50_ dose–response curves are shown as normalized values to the top and bottom values in Graphpad Prism. The reported values in the figures are shown as mean ± standard error of the mean (SEM).

## Results & discussion

### Discovery of ERK active-site peptides for isoform-selective activity-based profiling

Mammalian ERK1 and ERK2 show high sequence homology (>80% identity^6^,^15^) and contain largely indistinguishable substrate binding sites where 22 out of the 23 residues are identical between isoforms.^20^ Our goal was to use covalent activity-based probes to map features of ERK active-sites under native conditions that could be leveraged for isoform-specific activity-based profiling using chemoproteomic methods.^45^–^47^ For these experiments, we chose to use ATP acyl phosphate activity-based probes because these probes balance substrate recognition (*via* the ATP binding element) with proteome reactivity through the electrophilic acyl phosphate group.^39^,^40^ Previous studies demonstrated that the tuned specificity/reactivity of these probes can reveal active features that are not captured by crystallography^48^ or conventional biochemical substrate assays.^41^,^42^ ATP acyl phosphates covalently modify amino groups of conserved lysine side chains in active-sites of kinases and other ATP-binding proteins for global proteomic analysis^39^–^42^ (Fig. 2C).

To probe native ERK active sites, we treated mouse brain proteomes with ATP acyl phosphates and performed quantitative chemoproteomic analysis following previously published methods^41^,^42^ and as depicted in Fig. 2C. In brief, mouse brain soluble proteomes were pretreated with DMSO vehicle or free ATP (1 mM), respectively, prior to addition of ATP acyl phosphate (10 μM ATP probe, 30 min) to label active-site lysines. ATP competition was included to confirm probe labeling at kinase active sites. After probe labeling, proteomes were digested with trypsin/Lys-C proteases and desthiobiotin-modified peptides were enriched by avidin affinity chromatography and analyzed by LC-MS/MS to identify active-site peptides from kinases and other ATP-binding proteins^39^–^42^ (see Experimental section for additional details on LC-MS analyses).

**Fig. 3:**
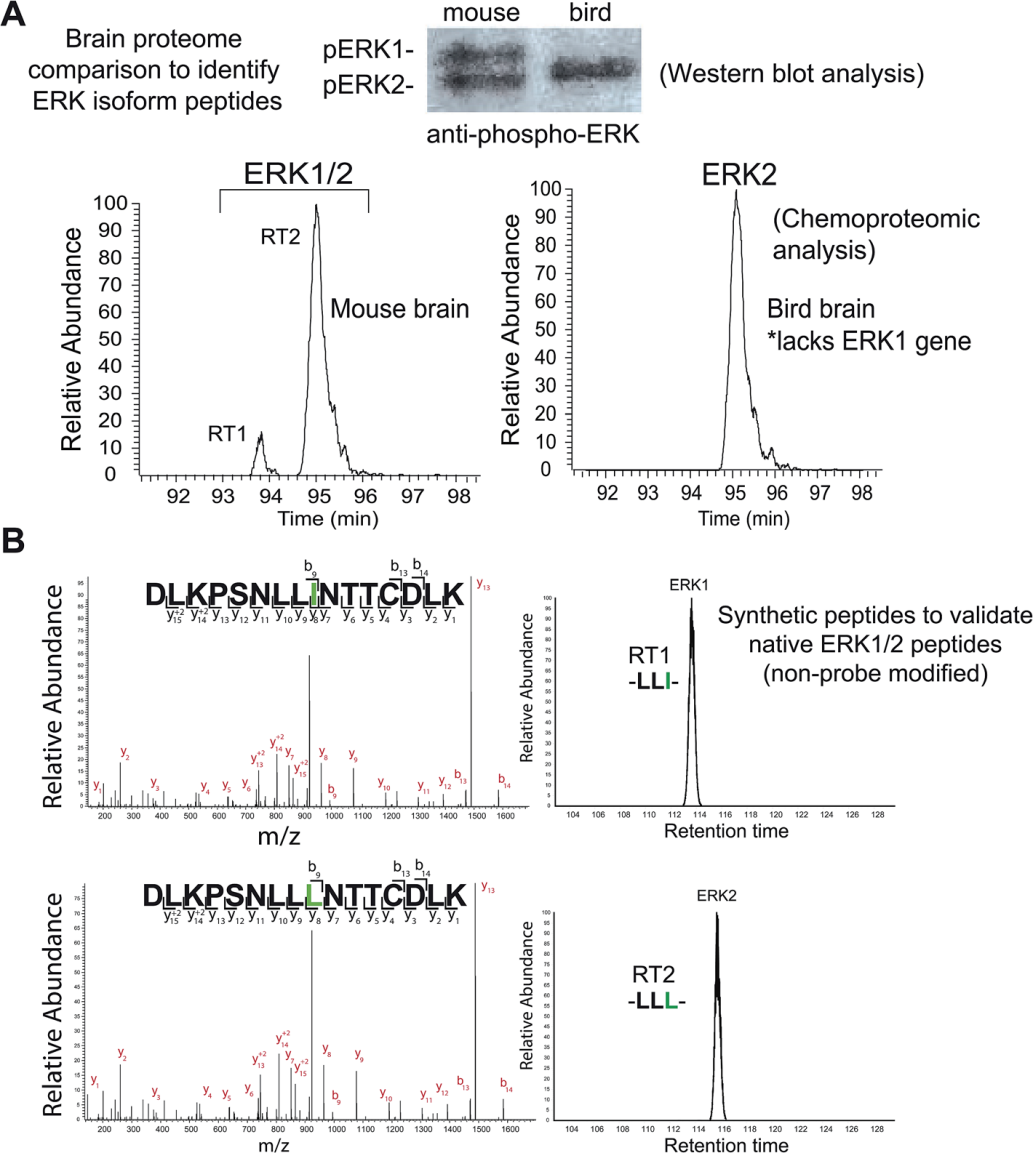
Discovery and identification of isomeric active-site peptides of mammalian ERKs. (A) Comparison of mouse and bird (*Taeniopygia guttata*) brain proteomes by chemoproteomic and western blot analyses. Birds lack the ERK1 gene, which enables distinction between the identities of ERK peptides detected from the mouse brain proteome. The elution time for the ERK2 peptide identified from bird brain matched the late-eluted peptide in mouse brain, which supports RT1 and RT2 as ERK1 and ERK2, respectively. MS2 fragmentation spectra of the bird brain ERK2 peptide was identical to that of the mouse brain ERK1 and ERK2 counterparts (see Fig. S2†). The presence of a single ERK in bird brain proteomes was confirmed by western blot analysis. (B) Synthetic peptides corresponding to non-probe modified ERK1 and ERK2 active site-sequences, differing by isoleucine (I) *versus* leucine (L), were analyzed to confirm elution profiles. The identical MS2 fragmentation spectra of the synthetic ERK1 (-LLI-, top) and ERK2 (-LLL-, bottom) peptides and similar LC elution profile are consistent with the results obtained for endogenous mouse brain ERK1 and ERK2 probe-modified peptides (see Fig. 2). Green amino acids highlight isoleucine/leucine isomers in ERK active site-sequences.

**Fig. 4:**
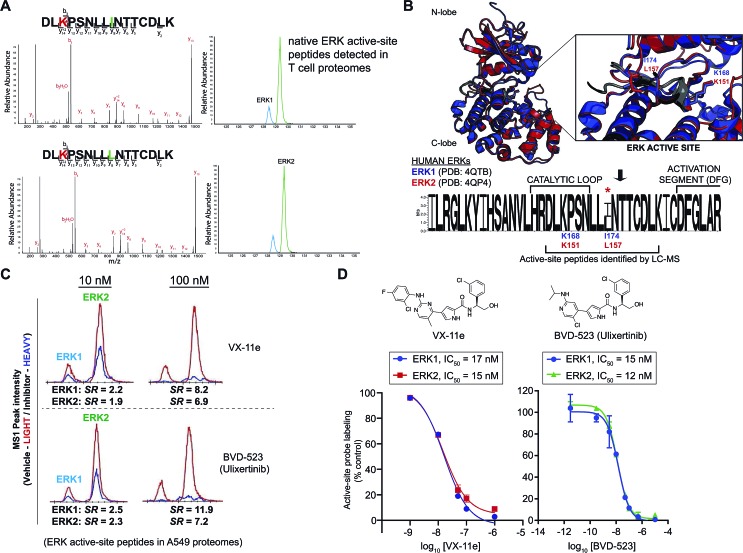
Enzyme and inhibitor profiling of native ERK isoforms by chemoproteomics. (A) Chemoproteomic analysis of primary human CD4+ T cells. Native ERK1 and ERK2 activity is detected by chromatographically-resolved, active-site peptides with identical MS2 spectra that recapitulates LC-MS findings from brain proteomes in Fig. 3. We obtain similar LC-MS data across diverse cell types as shown in Fig. S1,† highlighting use of this approach as a general activity assay of native ERKs. (B) Structural alignment of human ERK1 (PDB ID: ; 4QTB, blue) and ERK2 (; 4QP4, red) as described in the Experimental section. ERK1 and ERK2 exhibit high structural homology and near identical substrate binding sites (inset), which contains the catalytic lysine (ERK1 – K168; ERK2 – K151) that is probe-modified. Sequence logo analysis of LC-MS active site-peptide sequences across 6 mammalian species (human, cow, rodent, monkey, and pig) illustrates complete sequence homology with the exception of the single isoleucine/leucine isomer (ERK1 – I174; ERK2 – L157) that distinguishes ERK1 and ERK2. (C) Extracted ion chromatograms (EICs) showing inhibitory activity (determined by SILAC ratios, *SRs*) of VX-11e and BVD-523 against native ERK1 and ERK2 in A549 proteomes treated with vehicle (light) or compounds (heavy; 10 and 100 nM). (D) Dose–response curves from SILAC analyses to estimate potency of VX-11e and BVD-523 in A549 proteomes: VX-11e: ERK1 – IC_50_ = 17 nM (95% confidence interval (CI) of 12–24 nM), ERK2 – IC_50_ = 15 nM (95% CI of 10–23 nM); BVD-523: ERK1 – IC_50_ = 15 nM (95% CI of 11–20 nM), ERK2 – IC_50_ = 12 nM (95% CI of 11–15 nM).

We identified two closely eluting probe-labeled peptides that shared identical precursor (MS1) mass to charge ratios (*m*/*z* 1021.053, Δ*m*/*z* < 5 ppm) that were ambiguously matched to either ERK1 or ERK2 (RT1 and RT2 peptides, Fig. 2D). Inspection of MS/MS fragmentation (MS2) spectra revealed identical fragment ions including those corresponding to the modified lysine residue for early (RT1, Fig. 2E) and late-eluted ERK peptides (RT2, Fig. 2F). Both probe-modified peptides were potently competed with free ATP as determined by reductions in MS1 peak intensities, which supports active site labeling with the ATP acyl phosphate probe (Fig. S1A†). To determine if these probe-modified peptides correspond to ERK1 and ERK2 or if they are modified peptides from a single ERK isoform, we analyzed brain proteomes from *Taeniopygia guttata*. Gene sequencing and biochemical analyses have revealed that bird lineages lack the *erk1* gene^20^ and thus the *T. guttata* proteome serves as a natural ERK1 “knockout” for comparative analyses to assign isoform identity to peptides based on elution times in our liquid chromatography-mass spectrometry (LC-MS) studies. Our LC-MS studies revealed a single ATP-sensitive probe-modified peptide detected in *T. guttata* brain proteomes (Fig. S1B†), which had fragmentation spectra (Fig. S2†) and elution times matching the late-eluted ERK peptide identified in mouse brain samples (Fig. 3A). To exclude the possibility of tissue- and species-specific effects, we also measured ERK peptides in primary human T cell proteomes and discovered the same chromatographically-resolved peptides with MS2 fragmentation patterns (Fig. 4A) and ATP sensitivity (Fig. S1C†) that matched results from brain proteomes (Fig. 3A). Collectively, our findings support the early and late-eluted peaks as ERK1 and ERK2 active-site peptides, respectively, that could be detected across different cells and tissues from diverse mammalian species.

### Analysis of synthetic peptide standards to validate ERK1 and ERK2 isomeric active-site peptides

Sequences for the putative ERK1 and ERK2 peptides mapped to a region containing the catalytic loop and activation segment of the ERK substrate binding site (Fig. 4B). Multiple sequence alignments revealed complete conservation of this region across species for each respective ERK isoform (human, cow, rodent, monkey, and pig, Table S1†). A single isoleucine/leucine isomer differentiates ERK1 (I174) and ERK2 active-site peptides (L157; amino acid numbering for human ERKs). LC-MS studies to discriminate leucine and isoleucine isomers in peptide sequences is typically accomplished with multistage fragmentation strategies and customized LC-MS methods^49^–^51^ because these amino acids cannot be distinguished by mass alone. Since we did not implement specialized LC-MS methods, we hypothesized that the ERK1 and ERK2 isomeric peptides are resolved and detected by reverse-phase LC-MS strictly based on differences in hydrophobicity of leucine and isoleucine.

To validate our hypothesis, we analyzed synthesized peptides with sequences matching ERK1 and ERK2 active-site tryptic peptides using the same LC-MS parameters employed for our chemoproteomic studies. Analysis of commercial synthetic peptides DLKPSNLLINTTCDLK (ERK1) and DLKPSNLLLNTTCDLK (ERK2) confirmed >95% purity for these peptides (Fig. S3A†). Comparison of retention times in our LC-MS analyses of synthetic ERK1 and ERK2 peptides (Fig. 3B) recapitulated the chromatographic elution profile observed for probe-modified active-site peptides detected in native proteomes, *i.e.* ERK1 peptides have earlier elution times compared with ERK2 peptides (Fig. 4A and S1†). We also analyzed mixtures of synthetic ERK1 and ERK2 peptides to dismiss potential LC variability between sample runs as the explanation for the observed chromatographic behavior. We analyzed ERK1 and ERK2 synthetic peptides in 10 : 1 and 1 : 10 mixtures and used signal intensities of precursor MS1 peaks to correlate peptide identity and elution profiles (Fig. S3B†). Our results show the presence of 2 distinct peptides with MS1 intensities that match the ratio of mixed synthetic ERK1/ERK2 peptides (Fig. S3B†) and confirm elution profiles of endogenous ERK1 and ERK2 active-site peptides observed in proteomes (Fig. 4A and S1†). Our findings constitute a novel demonstration of the capability of HPLC to separate I/L isomeric peptides in a complex proteomic sample. Importantly, we exploit this unique feature of ERK active sites to enable activity-based profiling of ERK1 independent of ERK2 (Fig. 2C).

### Chemoproteomic profiling of ERK1 and ERK2 activity across tumor cell panels

We envision chemoproteomic profiling of native ERK1 and ERK2 as a universal activity assay that would complement widely-used cell biological assays^6^,^34^ to gain insights into ERK signaling in tumor cell biology (Fig. 1). To broadly evaluate ERK activity across a panel of tumor cells, we employed stable isotope labeling with amino acids in cell culture (SILAC^52^) to enable quantitative proteomic analysis using ATP acyl phosphate probes as previously described.^41^,^42^ Tumor cells were cultured in isotopically light and heavy amino acids (K, R), lysed, and labeled proteomes used for chemoproteomic analysis. In brief, light and heavy lysates were treated differentially with DMSO vehicle or free ATP (1 mM) respectively, prior to addition of ATP acyl phosphate to label active site lysines. After probe labeling, light and heavy proteomes were combined, digested with trypsin/Lys-C protease, and desthiobiotin-modified peptides enriched by avidin affinity chromatography and analyzed by LC-MS/MS to identify and quantify isotopically tagged active-site peptides from ERK1 and ERK2 (Fig. 2C).

Using our quantitative chemoproteomic assay, we detected native ERK1 and ERK2 activity across a panel of non-small cell lung cancer (NSCLC) and small cell lung cancer (SCLC) cell lines (Fig. S1D†). We profiled cell lines that differed by tumor type and mutational status. We chose H1650 and A549 as our NSCLC cell models to compare ERK activity in cells with differing EGFR receptor mutations. H1650 cells express EGFR receptors that contain activating mutations in the kinase domain (exon 19 deletion E746-A750 (ref. 53)) of this receptor tyrosine kinase. A549 cells express wild-type EGFR but harbor mutant KRAS (G12S).^54^ We also included H82 cells^55^ in our studies to evaluate ERK activity in small cell lung cancer (SCLC) cells harboring mutations in MEK2.^56^ Akin to results from analysis of brain proteomes, both ERK1 and ERK2 probe-modified peptides were highly competed with ATP treatment as determined by SILAC ratios (*SR*) of MS1 chromatographic peak areas >5 in DMSO/ATP comparisons (Fig. S1D†). From our activity measurements across lung cancer proteomes, we calculated that ERK1 and ERK2 contribute ∼20% and ∼80% of the total endogenous ERK activity, respectively (Fig. S1D†). Our findings match those of previous work showing higher ERK2 expression due to a stronger *erk2* promoter and correlate nicely with protein expression data measuring ratios of native ERK1 and ERK2 proteins in cell lines.^22^

In summary, we demonstrate that our ERK chemoproteomic assay is capable of independent profiling of ERK1 and ERK2 activity across tumor cells (Fig. S1D†), primary cells (Fig. 4A), and tissues (Fig. 3A). Our assay should serve as a universal ERK activity assay given the complete conservation of measured active-site peptides across a wide range of species (sequence logo analysis shown in Fig. 4B and Table S1†). Future studies are needed to determine whether the ratio of ERK1 and ERK2 activity is perturbed under different activation paradigms and from different microenvironments *in vivo* (*e.g.* from patient tumors).

### Inhibitor profiling of native ERK1 and ERK2

Next, we asked whether we could use our ERK chemoproteomic assay to evaluate specificity of inhibitors against native ERK1 and ERK2. Isoform specificity of ERK inhibitors are conventionally determined using assays of recombinant ERK1 and ERK2 (ref. 31–33) (Fig. 2A). Activity of inhibitors against endogenous ERK in cellular and animal models is largely ascribed to both isoforms (denoted by ERK1/2, Fig. 2B) based on the assumption that overlapping substrate specificity observed with recombinant proteins will translate to biology observed with native protein.^6^,^19^ Here, our goal was to determine whether our chemoproteomic assay could profile inhibitor activity against native ERK1 and ERK2 independently and directly in complex proteomes (Fig. 2C).

Several optimized ERK1/2 inhibitors have been reported to date, and the selectivity of these compounds against ERK isoforms have been determined largely for recombinant proteins.^43^,^44^,^57^–^61^ For our studies, we selected the ERK inhibitor VX-11e (*i.e.* Vertex-11e), which was originally discovered as a potent, selective, and orally bioavailable ERK2 inhibitor.^57^ Further studies demonstrated that VX-11e is a type-I kinase inhibitor with unique kinetic properties defined by slow dissociation rates^31^,^61^ that results in potent inhibitor activity against both recombinant ERK1 and ERK2.^61^ It has not been determined if VX-11e exhibits ERK isoform specificity in native systems. To evaluate selectivity of VX-11e, A549 soluble proteomes were pretreated with compound at varying concentrations followed by labeling with ATP acyl phosphate probe and quantitative chemoproteomic analysis (Fig. 2C). Potent competition at ERK1 and ERK2 active sites was determined by SILAC ratios of MS1-extracted ion chromatograms in DMSO/VX-11e comparisons (*SR* > 5, Fig. 4C). We calculated the potency of VX-11e against endogenous ERK1 and ERK2 and found equivalent and potent inhibition based on calculated IC_50_ values (ERK1, IC_50_ = 17 nM, 95% confidence intervals (CI) of 12 to 24 nM; ERK2, IC_50_ = 15 nM, 95% CI of 10 to 23 nM; left panel, Fig. 4D). Our potency measurements of endogenous ERKs by VX-11e match previous findings of near-equivalent inhibition of purified ERK1 and ERK2 using biophysical kinetic binding assays.^61^

We also tested the ERK inhibitor BVD-523 (Ulixertinib^43^), which is in clinical trials for cancer therapy (NCT01781429, NCT02296242, and NCT02608229). Akin to VX-11e, Ulixertinib is a type-1 reversible, ATP-competitive inhibitor.^43^ Biochemical substrate and calorimetric assays of recombinant ERKs showed enhanced potency of BVD-523 for recombinant ERK2 compared with ERK1 (∼8-fold enhanced potency for ERK2 (ref. 43)). We determined that BVD-523 showed equipotent activity against endogenous ERK1 and ERK2 by chemoproteomics (ERK1, IC_50_ = 15 nM, 95% CI of 11 to 20 nM; ERK2, IC_50_ = 12 nM, 95% CI of 11 to 15 nM; right panel, Fig. 4D). While our assay format (*i.e.* displacement of probe-binding by inhibitors) for measuring BVD-523 potency is different than conventional substrate assays, our calculated ERK2 IC_50_ values for BVD-523 are comparable with potency estimates using reported biochemical assays (∼2 nM (ref. 57)). We propose the difference in isoform selectivity between methods is a reflection of assaying recombinant *versus* native kinase proteins; similar discrepancies have been observed for other clinical kinase inhibitors using chemoproteomic assays.^39^ In addition to BVD-523,^43^ several optimized ERK inhibitors including GDC-0994 (ref. 44) (NCT01875705, NCT02457793) and MK8353 (NCT02972034) are currently in clinical trials. Correlation of efficacy with selectivity data against native ERK isoforms should provide critical insights into the inhibition mechanism and determine whether isoform-selective or global ERK inhibitors are more efficacious cancer therapies.

### Clinical ERK inhibitors mediate tumor cell killing *via* equipotent inhibition of ERK1 and ERK2

**Fig. 5:**
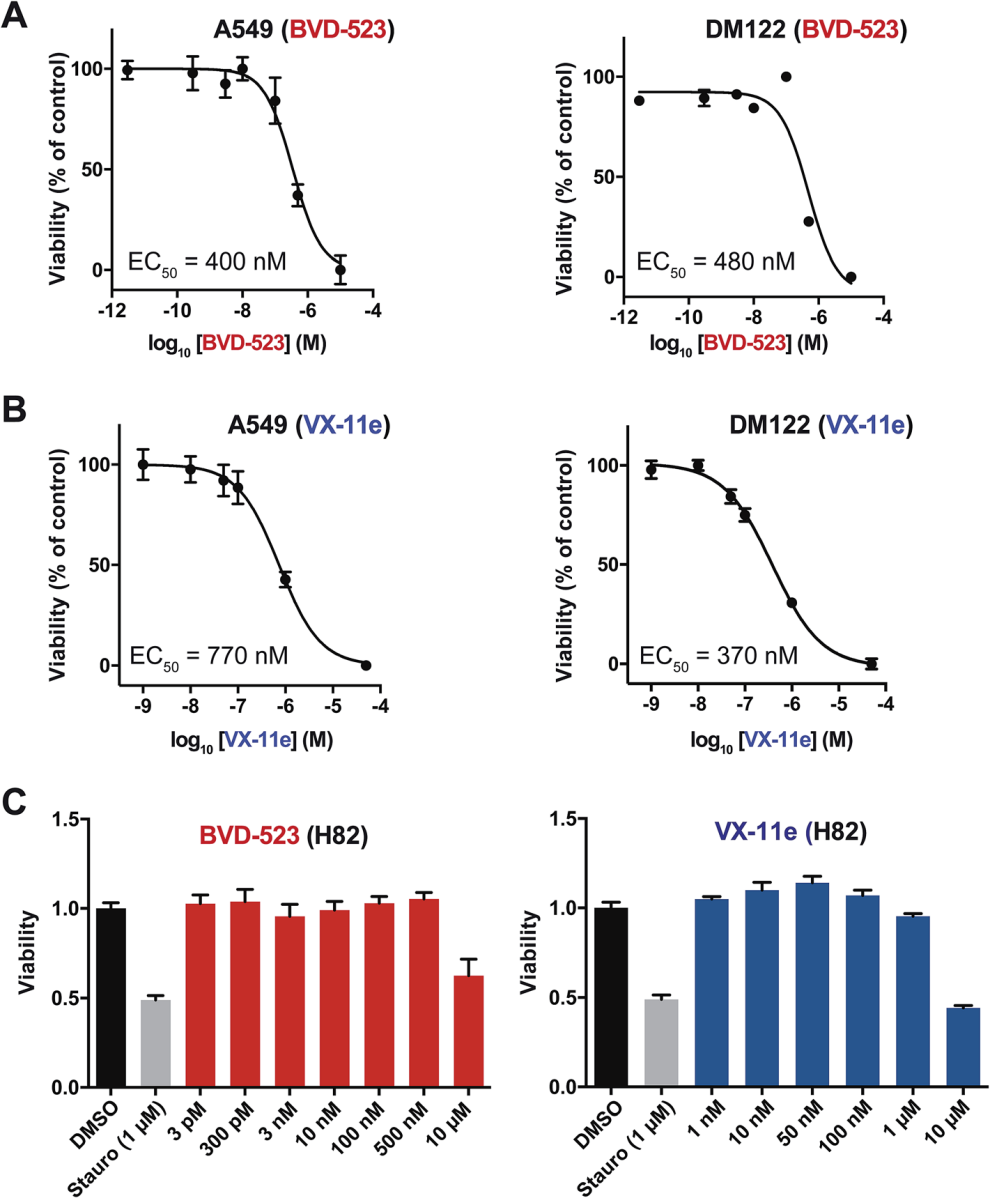
Cytotoxicity of ERK inhibitors in tumor cells. VX-11e and BVD-523 were tested for cytotoxic activity against A549 (NSCLC), DM122 (melanoma), and H82 (SCLC) cells. Cells were incubated with compounds at varying concentrations for 4 days followed by measurement of cell proliferation using established metabolic assays (WST-1). Dose–response curves were generated to evaluate cellular potency (EC_50_ values) of compounds. (A) The EC_50_ values for BVD-523 in cell lines were calculated as follows: A549, EC_50_ = 400 nM (95% CI of 200–750 nM); DM122, EC_50_ = 480 nM (95% CI of 270–870 nM). (B) The EC_50_ values for VX-11e in cell lines were calculated as follows: A549, EC_50_ = 770 nM (95% CI of 420–1400 nM); DM122, EC_50_ = 370 nM (95% CI of 250–540 nM). (C) H82 cells were tested with the same inhibitor concentrations used in (A) and (B). VX-11e and BVD-523 were less cytotoxic in H82 cells, and substantial blockade of cell proliferation was only observed at 10 μM concentrations. Staurosporine (Stauro; pan-kinase inhibitor) was included as a positive control for our cytotoxicity experiments.

Next, we measured viability of A549 (NSCLC), H82 (SCLC), and DM122 (melanoma) cells exposed to VX-11e and BVD-523 in order to correlate potency estimates by chemoproteomics (Fig. 4D) with cellular activity of compounds. Tumor cells were treated for 4 days at varying concentrations and cell proliferation monitored using established metabolic assays.^62^ We observed dose-dependent blockade of cell proliferation in A549 and DM122 cells treated with both BVD-523 (Fig. 5A) and VX-11e (Fig. 5B). Specifically, VX-11e and BVD-523 displayed comparable cytotoxicity in A549 and DM122 cells as judged by the measured effective concentrations (average EC_50_ values in the range of ∼400–700 nM, Fig. 5A and B). In contrast, H82 cells were largely resistant to VX-11e and BVD-523 treatments; substantial cell death only occurred in H82 cells treated at the highest concentration of VX-11e and BVD-523 tested (10 μM for both compounds, Fig. 5C).

**Fig. 6:**
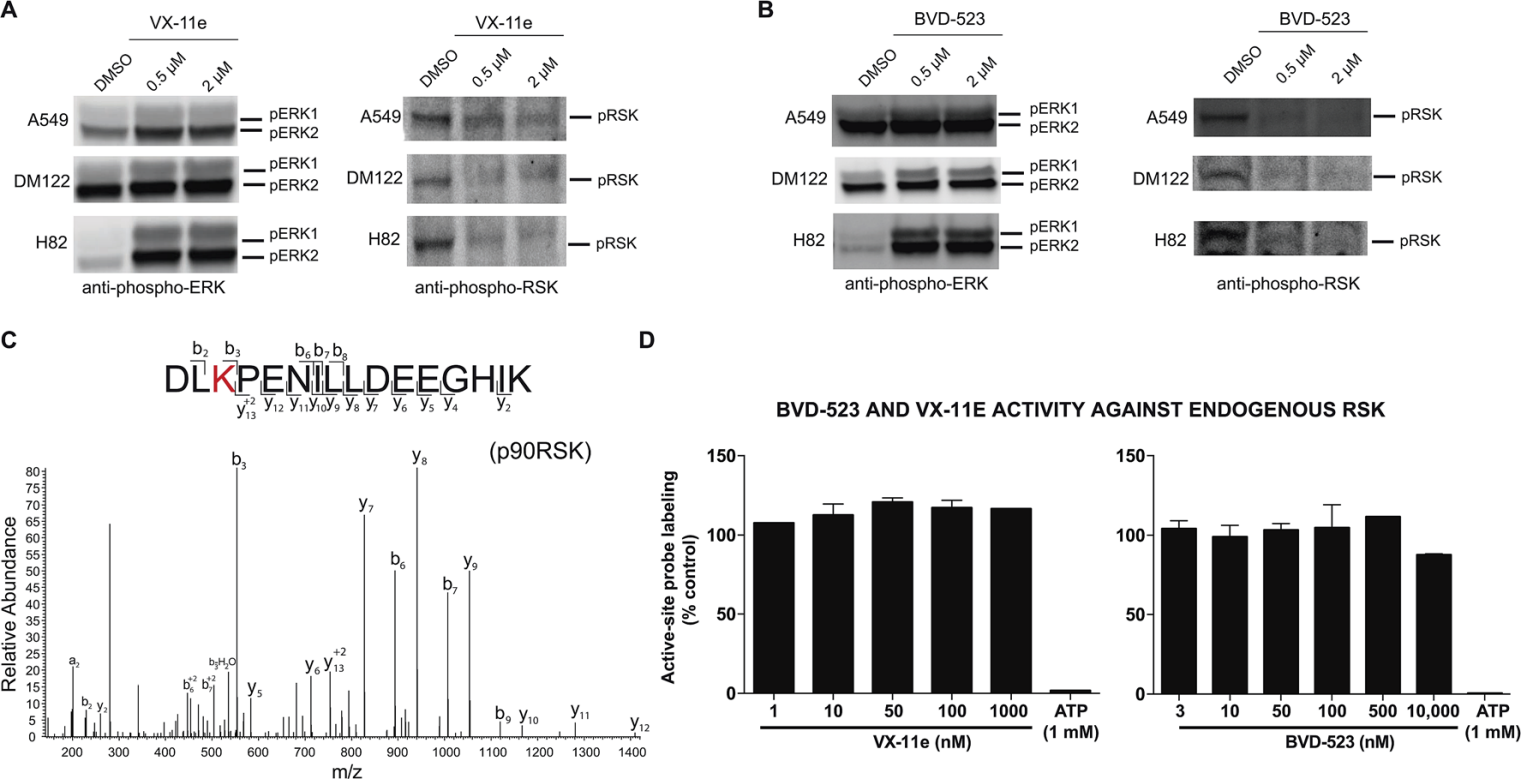
Cell biology of ERK inhibitor activity in tumor cells. Western blot analysis to determine phosphorylation status of ERK1/2 and RSK in A549, DM122, and H82 cells treated with VX-11e and BVD-523. Cells were treated with each compound at respective concentrations for 4 hours in serum free media. Samples were analyzed with antibodies against p-ERK (Thr202/Tyr204) and p-RSK (Thr359/Ser363). (A, B) VX-11e and BVD-523 showed inhibition of phosphorylation for p-RSK at 0.5 μM and 2 μM. A549 and DM122 showed a slight increase in phosphorylation for ERK1/2 when treated with VX-11e and BVD-523. In contrast, H82 showed a massive enhancement in ERK1/2 phosphorylation upon compound treatment. (C) MS2 fragmentation spectra of RSK active-site peptides from quantitative chemoproteomic analysis. (D) SILAC analyses confirmed that VX-11e and BVD-523 are not inhibitors of endogenous RSK in A549 proteomes. Sensitivity of RSK peptide to ATP competition confirmed active site-dependent probe labeling in our chemoproteomic studies. RSK peptide used for analysis is shared between RSK1, RSK2, and RSK3.

To gain further insights into the mechanism and compound effects on cellular signaling, we performed western blot analyses of ERK1/2 phosphorylation and the downstream substrate p90RSK,^63^ which are established biomarkers for evaluating compound activity against native ERK1/2.^43^ For these studies, cells were treated with vehicle or compound for 4 hours followed by western blot analysis of phosphorylated proteins. We observed mild increases in ERK1/2 phosphorylation in A549 and DM122 cells treated with VX-11e and BVD-523 at moderate (0.5 μM) and high concentrations tested (2 μM compounds, left panels; Fig. 6A and B). Our findings match reports of paradoxical increases in ERK1/2 phosphorylation in various tumor cells treated with VX-11e and BVD-523, albeit to a lesser magnitude than previously observed.^43^,^58^ In striking contrast, H82 cells displayed low basal ERK1/2 phosphorylation that was massively enhanced in VX-11e and BVD-523 treated cells (left panels, Fig. 6A and B). Reactivation of phospho-ERK by VX-11e and BVD-523 is likely due to feedback activation pathways as previously described.^16^ For all 3 cell lines, enhancement of ERK1/2 phosphorylation was achieved at 500 nM of inhibitor with no further increase in phosphorylation at higher compound concentrations (left panels; Fig. 6A and B).

**Fig. 7:**
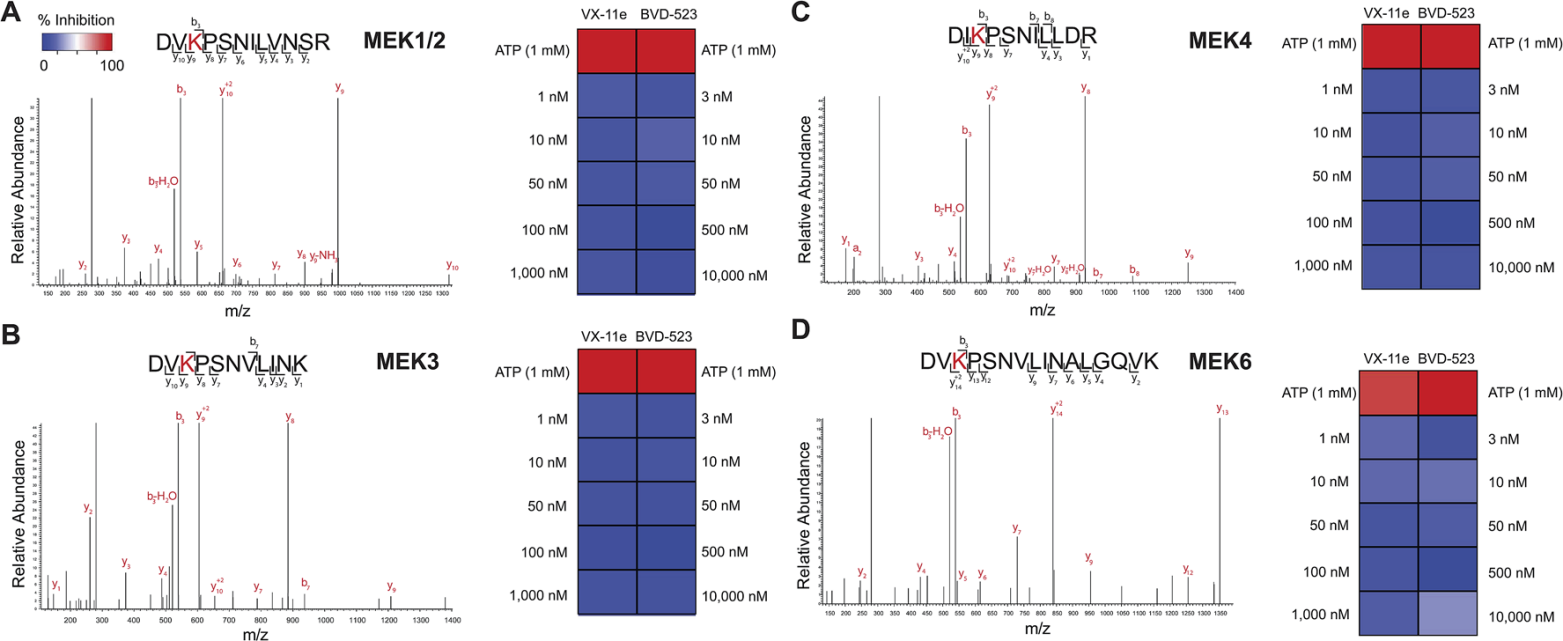
Selectivity of ERK inhibitors against upstream MAPK regulators. (A) MEK1/2 are kinases that phosphorylate ERK1 and ERK2. SILAC analyses in A549 proteomes confirmed that VX-11e and BVD-523 are not inhibitors of endogenous MEK1/2. (B–D) Activity of VX-11e and BVD-523 was tested against additional MEK kinases and found to be inactive. Sensitivity of MEK kinase active-site peptides to ATP competition (1 mM) confirmed active site-dependent probe labeling. Lack of inhibitory activity of VX-11e and BVD-523 against endogenous MEKs supports on-target activity of compounds against native ERK1 and ERK2.

We also demonstrated that VX-11e and BVD-523 treatments blocked phosphorylation of the downstream ERK substrate p90RSK (right panels, Fig. 6A and B). Akin to effects observed for ERK1/2, maximal blockade of RSK phosphorylation occurred at 500 nM of compound with no appreciable increases in inhibitor activity at higher concentrations of VX-11e or BVD-523 (right panels, Fig. 6A and B). We also used quantitative chemoproteomics to show that VX-11e and BVD-523 are not inhibitors of endogenous RSK (RSK1/2/3 active-site peptide detected in A549 proteomes, Fig. 6C and D). The lack of RSK inhibition using VX-11e and BVD-523 was maintained even when tested at concentrations ∼1000-fold higher (10 μM, Fig. 6D) than potency values observed for ERK (average IC_50_ value of ∼15 nM, Fig. 4D). These studies were important to confirm that the observed cell biology is due to on-target inhibition of ERK1/2 and not off-target activity against RSK directly. We also confirmed that VX-11e and BVD-523 showed negligible activity against a panel of endogenous MEKs including direct regulators of ERKs (MEK1/2, Fig. 7A) and other MEKs involved in MAPK signaling (MEK3, MEK4, and MEK6, Fig. 7B–D). For all kinases tested, we confirmed quantitation of active sites by demonstrating near complete blockade of probe labeling upon pretreatment with free ATP (1 mM, Fig. 7 and S4†). Collectively, our studies support that VX-11e and BVD-523 mediate cell biological effects through on-target and equipotent blockade of ERK1 and ERK2 activity.

From our chemoproteomic studies, we estimate ∼90% blockade of both native ERK1 and ERK2 activity with treatment of ∼0.5–1 μM of VX-11e and BVD-523 (Fig. 4D). The concentrations that provide near-complete blockade of ERK1 and ERK2 are highly correlated with maximal cell biological response (RSK phosphorylation, right panels; Fig. 6A and B) and cytotoxicity observed in cell proliferation assays (Fig. 5A and B). Based on our chemoproteomic and cell biological findings, we propose a model whereby >90% blockade of native ERK1 and ERK2 is required for cellular activity of BVD-523 and VX-11e. Although our potency estimates of BVD-523 are substantially higher than those reported from recombinant ERK assays,^43^ we believe the inhibitor competition profiles against native ERKs more closely mirror the cellular activity of ERK inhibitors. Discrepancies in potency estimates from *in vitro*/recombinant kinase assays and cellular activity of compounds have also been observed with RAF inhibitors.^39^ The authors demonstrated that *in vivo* activity of RAF inhibitors was highly consistent with inhibitory profiles against native RAF proteins determined by chemoproteomics, which was attributed to differences in behavior of recombinant and native RAF protein and not simply due to differences between assay format.^39^

## Conclusions

ERKs are critical nodes in cellular signaling and currently the focus of drug discovery efforts to battle resistance mechanisms observed in clinical agents targeting the MAPK pathway.^7^–^16^ Questions remain in the field regarding the contribution of ERK1 and ERK2 isoforms to cell signaling under physiological and pathological conditions.^19^ Consequently, the efficacy of isoform-selective *versus* global ERK inhibitors for blocking oncogenic MAPK signaling has not been explored. New platforms capable of measuring native ERK activity in an isoform-specific fashion would enable development of isoform-selective inhibitors to probe ERK1 *versus* ERK2 function. The challenge is the high sequence homology of ERK1 and ERK2 isoforms^6^,^15^ as well as the nearly identical substrate binding sites.^20^

We introduce a chemoproteomic strategy to profile native ERK1 and ERK2 active-sites directly in complex proteomes. We discovered that a single isoleucine/leucine difference in ERK1 and ERK2 substrate binding sites was sufficient to differentiate isomeric peptides and enable isoform-specific activity-based profiling of native ERK (Fig. 2C and 4B). Importantly, we performed our proteomic studies in complex proteomes to retain post-translational modifications and conformational states that can be lost in analyses of recombinant proteins. The ability to distinguish isoleucine and leucine in peptide sequencing experiments is technically daunting and typically accomplished using multistage fragmentation strategies and customized LC-MS workflows.^49^–^51^ We demonstrate that our chemoproteomic strategy can achieve baseline resolution and quantitation of I/L-isomeric ERK active-site peptides using standard reverse-phase LC-MS configurations and data-dependent MS acquisitions used in proteomics^64^ (Fig. 3B and 4A). Our assay also provides the advantage of profiling selectivity of ERK inhibitors against other kinases detected in lysates in parallel with selectivity assessment against native ERK isoforms (Fig. 6C and 7).

Our chemoproteomic and cell biological findings reveal a correlation that supports >90% inactivation of both native ERK1 and ERK2 to explain cellular activity of VX-11e and Ulixertinib. From these studies, we also conclude that ERK1/2 phosphorylation is not a suitable biomarker of cellular activity of ERK inhibitors like VX-11e and BVD-523 because H82 cells that show greatest enhancement in ERK1/2 phosphorylation (left panels, Fig. 6A and B) are largely resistant to cytotoxic effects of compounds (Fig. 5C). In agreement with our findings, recent clinical trials of BVD-523 have selected phospho-RSK as a pharmacodynamic marker of response to ERK inhibitors (NCT02296242). Future studies are needed to determine the underlying signaling pathways responsible for enhanced phospho-ERK1/2 and resistance of H82 cells to ERK inhibitors. Finally, phosphorylation of ERK2 results in a massive enhancement in catalytic activity;^6^ future studies are needed to evaluate ATP acyl phosphate probe binding of native ERK2 as well as ERK1 under different phosphorylation states to enable targeting of ERK isoforms under various cell signaling states.

In summary, our chemoproteomic assay provides the advantage of direct assessment of inhibitor activity at native ERK active-sites that is not confounded by potential resistance pathways that result in reactivation of ERK. Integration of chemoproteomic and cell biological assays of ERK activity should prove valuable for guiding development of ERK inhibitors and selection of appropriate cellular biomarkers for evaluation in research and clinical settings.

## Conflicts of interest

The authors declare no competing financial interest.

## Supplementary Material

Supplementary information
