# Supplementary material for: Isoform-selective activity-based profiling of ERK signaling
Source: Chem Sci. 2018 Feb 6;9(9):2419–31. doi: 10.1039/c8sc00043c (PMC5909473; doi:10.1039/c8sc00043c)
Supplement: Supplementary file 1 [file SC-009-C8SC00043C-s001.pdf]

Electronic Supplementary Material (ESI) for Chemical Science.
This journal is © The Royal Society of Chemistry 2018

SUPPLEMENTARY INFORMATION

Isoform-selective activity-based profiling of ERK signaling

Myungsun Shin[1], Caroline E. Franks[1], and Ku-Lung Hsu[1,2]*

[1]Department of Chemistry, University of Virginia, Charlottesville, Virginia 22904, United States
[2]Department of Pharmacology, University of Virginia, Charlottesville, Virginia 22908, United States

SUPPLEMENTAL METHODS

Sample preparation for quantitative LC-MS analysis using ATP acyl phosphates.
HEK293T and A549 cells were lysed using kinase buffer (10 mL DPBS (GE healthcare), protease inhibitor (Thermo Fisher Scientific), and 20 mM $MgCl_2$) and centrifuged at 100,000 x *g* for 45 min. Supernatant was separated from the pellet and protein concentration standardized to 1 mg/mL using kinase buffer. 'Light' proteomes and 'heavy' proteomes were pre-treated with DMSO and compound stock in DMSO, respectively. Samples were incubated at 25 °C for 30 min. Desthiobiotin ATP acyl phosphate nucleotide probe was dissolved in ddH2O and added to each sample at final concentration of 10 µM and incubated at 25 °C for 30 min. After the incubation, light and heavy proteomes were mixed and excess probe was extracted twice using $MeOH/CHCl_3/H_2O$ (4:1:3, v/v/v). Protein pellets were resuspended using sonication in MeOH and centrifuged at 17,000 x *g* for 5 min. Pellets were resuspended in 10 M Urea/25 mM ammonium bicarbonate in ddH2O. Reduction and alkylation of proteins were performed by reaction with dithiothreitol (10 mM final concentration) for 15 min at 65 °C, followed by iodoacetamide (40 mM final concentration) for 30 min in the dark. Excess reagents were extracted as described above and resulting pellets were resuspended in 25 mM ammonium bicarbonate. Protein samples were digested using Trypsin/Lys-C (Promega, 0.5 µg/µL) for 3 hrs at 37 °C. Digested samples were incubated with Avidin-agarose beads (Thermo Scientific Pierce) for 1 hr at 25 °C. Beads were washed 3 times each with 25 mM ammonium bicarbonate in dd H_2O. Probe labeled peptides were eluted using 50/50 CH_3CN/ddH2O with 0.1% formic acid. Peptides were dried by vacuum centrifugation and reconstituted in 50 µL of dd H_2O containing 0.1% formic acid in (LC mobile phase A) for LC-MS analysis.

LC-MS/MS analysis of proteomics samples.
The peptide samples were analyzed by LC-MS. An integrated autosampler-LC (Ultimate 3000 RSLC nanoSystem, Dionex) was used to load the peptides onto a trap column (Nano-Trap, Thermo Scientific, 2 cm, 5 µm C18) and washed for 2 min with 99% A (0.1% formic acid/H_2O)/1% B (80% CH_3CN, 1% formic acid). The peptides were eluted from the trap column and through a homemade nanocapillary analytical column (20 cm, 5 µm C18 packed in 360 µm o.d. x 75 µm i.d. fused silica), with an integrated electrospray tip, by reverse-phase LC (A: 0.1% formic acid; B: 80% CH_3CN, 0.1% formic acid) with the following gradient: 0-2 min 1% B, 400 nL/min; 2-144 min to 95% B, 300 nL/min; 144.1-180 min 1% B, 400 nL/min. The eluted peptides were electrosprayed into an Orbitrap Q Exactive Plus mass spectrometer (Thermo Scientific), which was operated with a top 10 data-dependent acquisition method that consisted of one full MS1 scan (375 - 1,500 m/z) followed by 10 MS2 scans of the most abundant ions recorded in the MS1 scan. Data analysis was accomplished using the IP2 (Integrated Proteomics Applications) software package, in which RawConverter was used to generate searchable MS1 and MS2 data from the .raw file followed by using the ProLuCID algorithm (publicly available at http://fields.scripps.edu/downloads.php) to search the data against a modified human protein database (UniProt human protein database with rat DGKs, angiotensin I and vasoactive intestinal peptide standards; 40,660 proteins) with the following parameters: static carbamidomethyl modification of cysteine (+57.0142 Da), differential modifications corresponding oxidized methionine (+15.9949 Da) and desthiobiotin-labeled lysine residues (+196.1212 Da), added masses of the SILAC "heavy"-labeled amino acids (+10.0083 Da for R, +8.0142 Da for K), and trypsin enzyme specificity with 2 missed cleavages. The resulting MS2 spectra-peptide sequence matches were assembled into protein identifications and filtered using DTASelect 2.0 using the --mass, --modstat, and --trypstat options with a 1% peptide FDR. ProLuCID search parameters were configured to allow for ambiguous identification of leucine/isoleucine in peptides (-a true). mzIdent files corresponding to searches were generated in IP2-Integrated Proteomics Pipeline, mzXML spectra data were extracted from the raw file using RawConverter, and uploaded into Skyline-daily[1] to determine SILAC ratios (SR) of light/heavy (vehicle/compound treated) peptides. Peptides used for analysis were assessed for quality in Skyline by the following criteria: isotope dot-

product (iDOTP) ≥ 0.8, ratio dot product (rDOTP) ≥ 0.8, and singletons (i.e. heavy-labeled peptide not detected) defined by L/H ratios > 20 were set to 20. Dot-product values are measures of similarity between the precursor peak area and expected isotope distribution (iDOTP) and between the light and heavy peak area (rDOTP) as calculated in Skyline and described previously[2, 3]. Probe-modified peptide reported in figures were manually inspected (MS1 and MS2 raw data).

SUPPLEMENTARY FIGURES

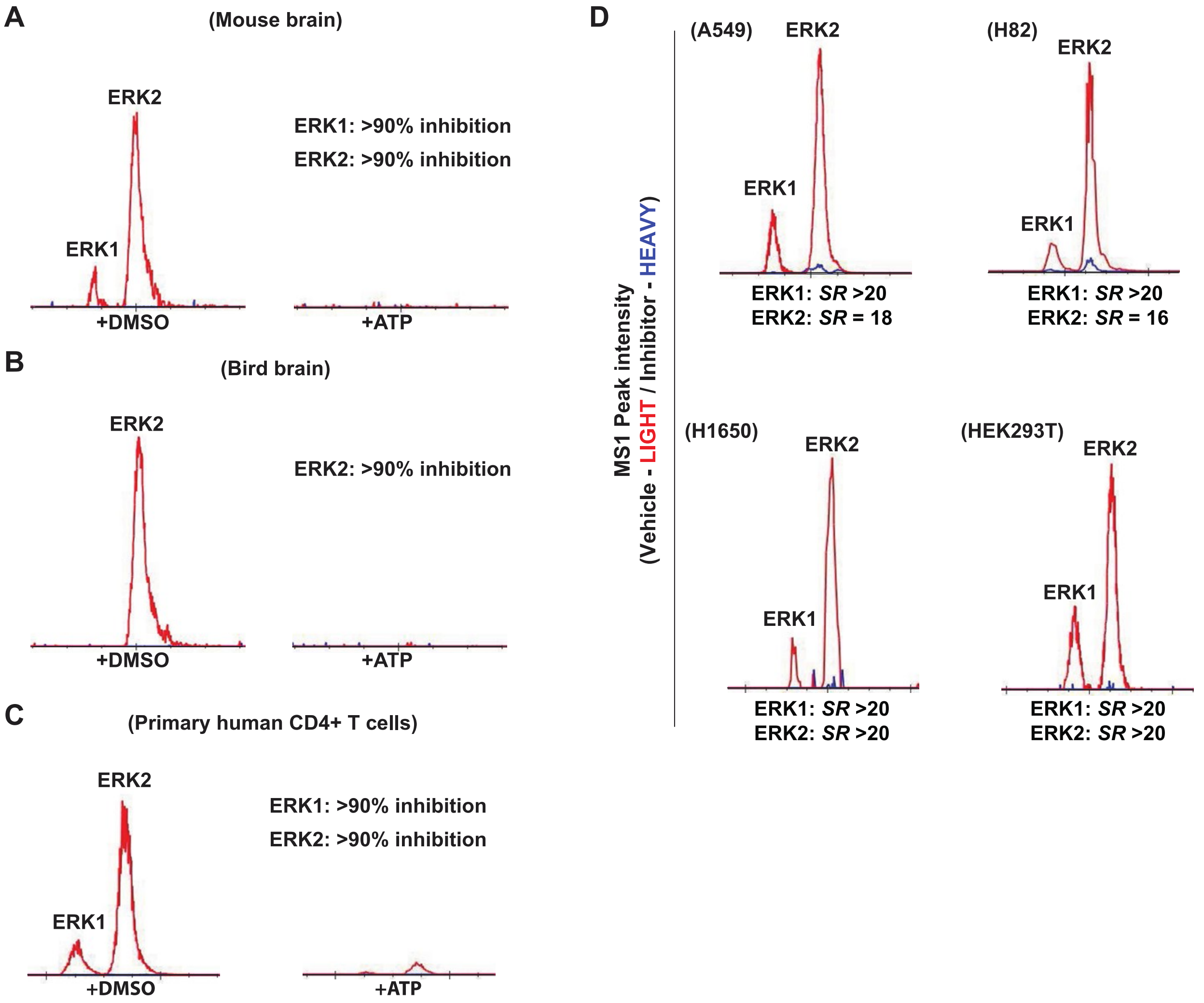

Figure S1. **Extracted ion chromatograms showing ATP sensitive ERK active-site peptides**. Sensitivity of native ERK1 and ERK2 active-site peptides to ATP competition shows specific and activity-dependent labeling in tissue (A, B), primary cell (C), and tumor cell proteomes (D). For brain and CD4+ T cell proteomes, pretreatment with free ATP (1 mM) resulted in >90% reductions in MS1 peak intensities of ERK1 and ERK active-site peptides. For tumor (A549, H1650, and H82) and HEK293T cells, quantitation of ATP sensitivity was determined by SILAC ratios of light and heavy proteomes pretreated with DMSO or free ATP (1 mM), respectively, prior to mixing and chemoproteomic analysis. A SILAC ratio (*SR*) >20 denotes a singleton, which indicates near-complete blockade of active-site probe-labeling with ATP pretreatment. Chemoproteomic analysis was carried out as described in previous studies[2, 3].

MS/MS FRAGMENTATION SPECTRA

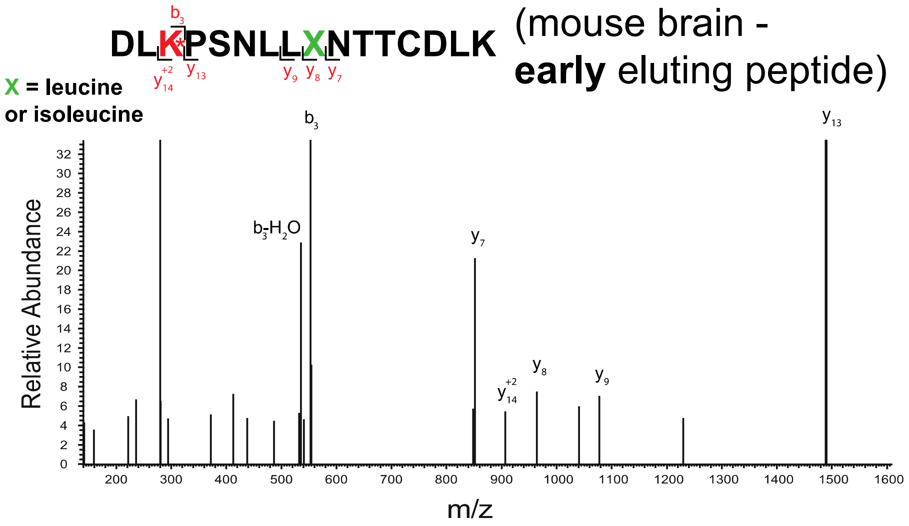

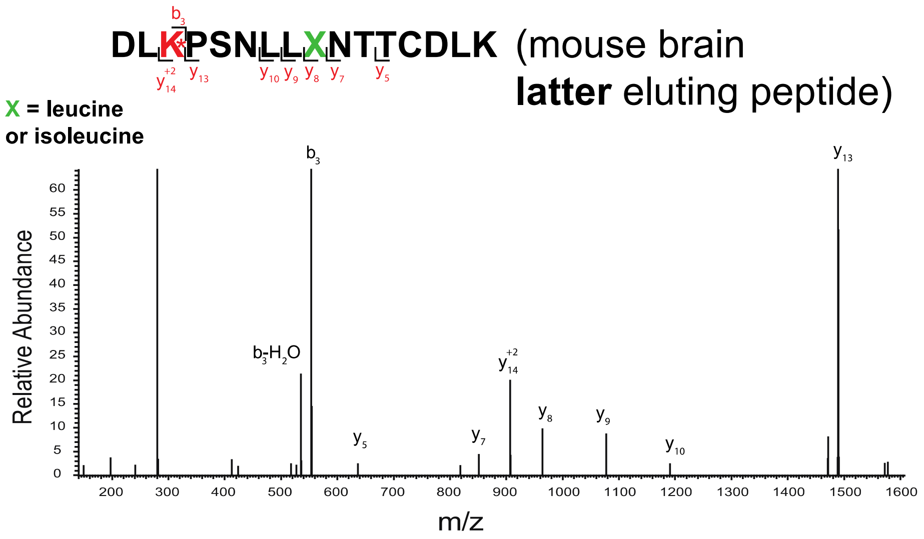

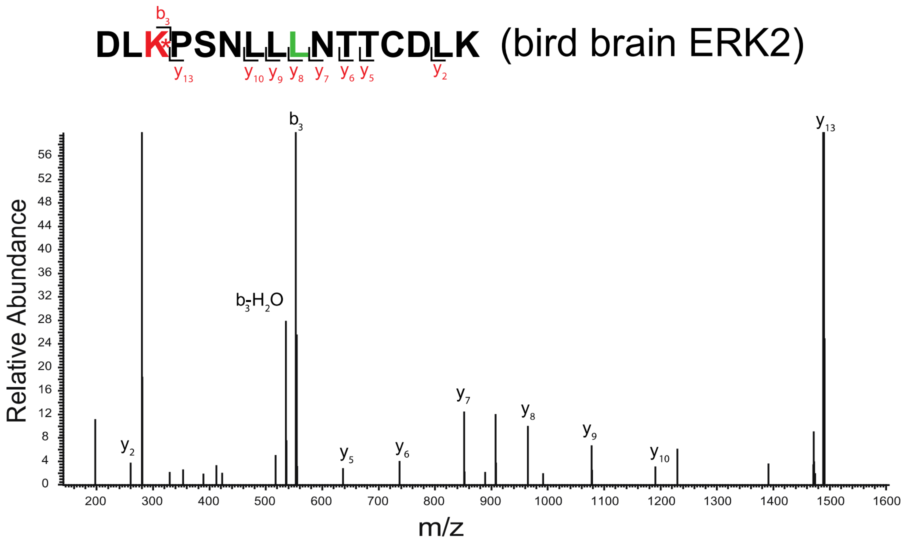

Figure S2. **Identical MS2 fragmentation spectra of ERK peptides identified in mouse and bird brain proteomes**. The result shows the fragmentation patterns observed in the early-eluted (RT1) and later-eluted (RT2) peaks of ERKs from mouse brain proteome are identical. Based on chemoproteomic analysis of bird brain proteomes, we demonstrated that the ERK2 active-site peptide has an elution time (LC retention) and MS2 fragmentation spectrum that match the later-eluted RT2 peak/peptide from mouse brain proteomes. These results support RT1 and RT2 as ERK1 and ERK2, respectively, in mouse brain proteomes.

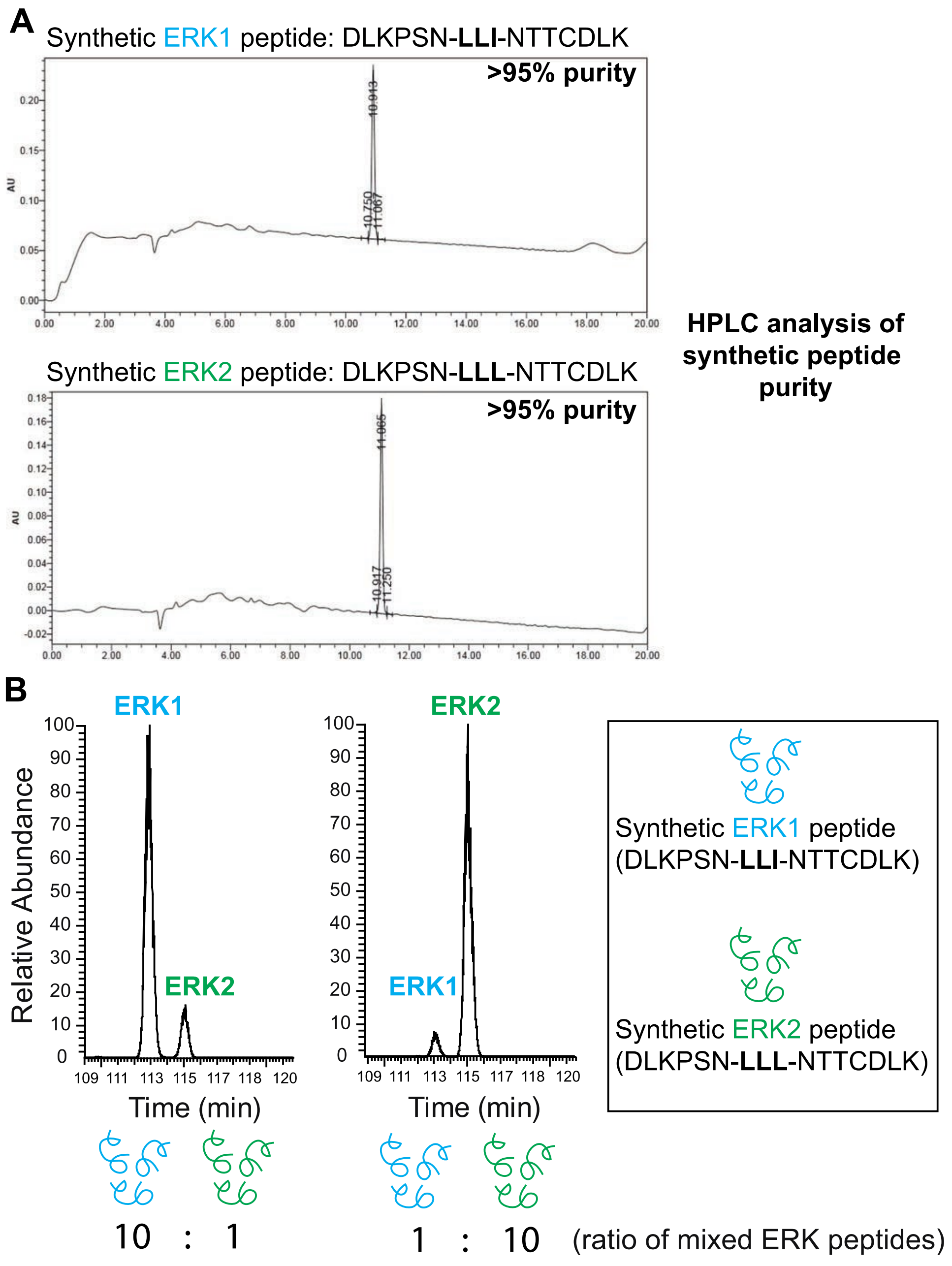

Figure S3. **Validation of elution profiles of synthetic peptides corresponding to ERK1 and ERK2 active-site peptides**. (A) Purity of synthetic peptides by HPLC analysis as described in the **Experimental section**. (B) Synthetic peptides for ERK1 and ERK2 are mixed at 10:1 (200 femtomole of DLKPSN-LLI-NTTCDLK peptide to 20 femtomole of DLKPSN-LLL-NTTCDLK peptide) and 1:10 (20 femtomole of DLKPSN-LLI-NTTCDLK peptide to 200 femtomole of DLKPSN-LLL-NTTCDLK peptide) ratios for each sample. The MS1 EICs shown for the doubly-charged peptides (m/z 922.992, $\Delta m/z < 5$ ppm) show that the ERK1 and ERK2 peptides were detected with expected relative abundances. Identical MS2 spectra shown in **Figure 3B** confirm that the single isoleucine/leucine difference between ERK1 and ERK2 active-site peptides can be distinguished by different LC retention times of parent peptides as measured by the MS1 data.

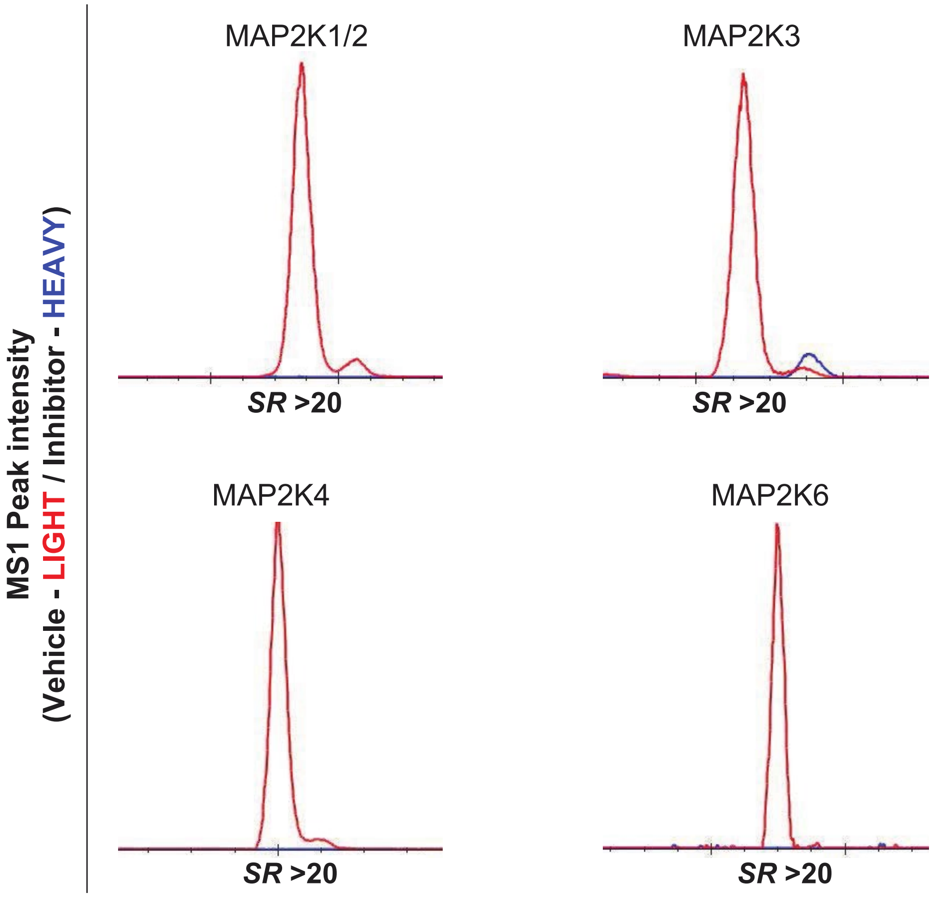

Figure S4. **Extracted ion chromatograms (EICs) showing ATP sensitive MEK active-site peptides**. A549 (lung carcinoma) cells were cultured in SILAC media (light – L, heavy – H) for quantitative chemoproteomics. The EICs are shown for 4 MEK active-site peptides (see **Figure 7**) with the light proteome (DMSO) in red and heavy (1 mM ATP) in blue. The results show that these 4 MEK active-site peptides are highly sensitive to ATP pretreatment as determined by SILAC ratios greater than 20 (L/H). Near-complete blockade of probe-labeling with free ATP supports detection of MEK active sites in our chemoproteomic studies.

REFERENCES

1. B. Schilling, M. J. Rardin, B. X. MacLean, A. M. Zawadzka, B. E. Frewen, M. P. Cusack, D. J. Sorensen, M. S. Bereman, E. Jing, C. C. Wu, E. Verdin, C. R. Kahn, M. J. Maccoss and B. W. Gibson, *Mol Cell Proteomics*, 2012, **11**, 202-214.
2. C. E. Franks, S. T. Campbell, B. W. Purow, T. E. Harris and K. L. Hsu, *Cell Chem Biol*, 2017, **24**, 870-880 e875.
3. R. L. McCloud, C. E. Franks, S. T. Campbell, B. W. Purow, T. E. Harris and K. L. Hsu, *Biochemistry*, 2017, DOI: 10.1021/acs.biochem.7b00962.
